# Laser-Written Tunable Liquid Crystal Aberration Correctors

**DOI:** 10.1021/acsphotonics.3c00907

**Published:** 2023-08-25

**Authors:** Alec Xu, Camron Nourshargh, Patrick S. Salter, Chao He, Steve J. Elston, Martin J. Booth, Stephen M. Morris

**Affiliations:** Department of Engineering Science, University of Oxford, Parks Road, Oxford, OX3 1PJ, United Kingdom

**Keywords:** optical aberrations, liquid crystals, Zernike polynomials, spatial light modulator, laser writing, two-photon polymerization

## Abstract

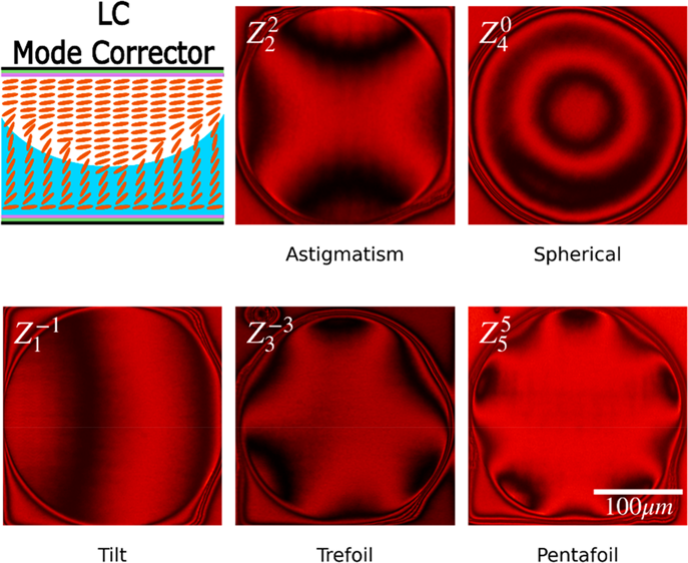

In this Article, we present a series of novel laser-written liquid crystal (LC) devices for aberration control for applications in beam shaping or aberration correction through adaptive optics. Each transparent LC device can correct for a chosen aberration mode with continuous greyscale tuning up to a total magnitude of more than 2π radians phase difference peak to peak at a wavelength of λ = 660 nm. For the purpose of demonstration, we present five different devices for the correction of five independent Zernike polynomial modes (although the technique could readily be used to manufacture devices based on other modes). Each device is operated by a single electrode pair tuned between 0 and 10 V. These devices have potential as a low-cost alternative to spatial light modulators for applications where a low-order aberration correction is sufficient and transmissive geometries are required.

## Introduction

Adaptive optics allows for aberration correction in imaging systems and is widely used to improve resolution and contrast to enhance image quality in fields as diverse as astronomy, microscopy, and ophthalmoscopy.^1^ In these fields, disturbances in the optical pathway, such as atmospheric turbulence in the case of astronomy and tissue refractive index variations in the case of microscopy, introduce artifacts in the detected image by distorting the phase profile of the propagating wavefronts. These distortions in the wavefront, which reduce imaging quality, can be described as a combination of linearly independent functions, such as the commonly used Zernike polynomial modes.

Aberration correction is usually performed using deformable mirrors, liquid crystal (LC) spatial light modulators (SLMs) or transmissive fluid-filled devices. Deformable mirrors use a flexible reflective mirror that is controlled by a set of actuators. The actuators introduce distortions into the surface of the mirror, providing phase modulation of the reflected light. The high number of actuators needed to properly shape the wavefront greatly increases the cost of these devices, making them both prohibitively bulky and expensive for some optical systems.^2^

Transmissive fluid devices use a series of either electrostatic^3^ or piezo-electric^4^ actuators to deform a fluid-filled membrane device, allowing it to follow the shape of one or more aberration modes. These devices are becoming increasingly flexible, now able to generate up to seventh-order Zernike polynomials.^3^ However, they still require bulky electronics to operate, requiring dozens of electrodes for correct operation. They also require high operational voltages of as much as 250 V to create such modes at length scales of the order of the wavelength of light.

SLMs, on the other hand, are pixelated devices that modify the wavefront of light by adjusting the effective refractive index of each LC pixel element independently, which, in turn, adjusts the phase of light propagating through each pixel. Much like deformable mirrors, SLMs are generally expensive optical components that require many individual electrodes to pixelate the aperture of the modulated beam. Furthermore, most SLMs are reflective, as they rely on the use of a silicon back plane for the drive electronics and thus generally require beam folding optical arrangements, which increases both the complexity and the footprint of the optical system. The combination of bulk, price, and complexity of deformable mirrors, transmissive fluid-based waveplates, and SLMs can generally make the devices prohibitively expensive for adaptive optics applications that require only the correction of a few relatively low order modes. Thus, for many imaging applications, a new type of functional device would be desirable: one that harnesses the tuning of the LC with a transmissive configuration and that does not have the need for bulky electronics.

The continuous tunable nature of the refractive index of nematic LCs has made them particularly attractive for phase control devices, with new developments in topological manipulation of these LCs increasing application across both imaging and communication.^5−7^ These developments are particularly attractive for tunable lenses for polarized light, providing a means of continuously controlling the focal length. While some lenses, such as the ones developed by Sato et al.,^8^ simply adjust the refractive index of the LC within an already curved substrate, others, such as those developed by Chen et al.,^5^ instead use a series of LC devices coupled with multiple electrodes to create a more complex lens configuration. There have also been great strides in recent years using sophisticated electrode patterns to recreate different commonly used lens shapes such as axicons as well as spherical and cylindrical lenses.^9^ Unfortunately, these techniques often struggle to recreate more complex Zernike modes such as the trefoil or pentafoil while still increasing the footprint and complexity of the device.

Polymerized nematic LCs can potentially enable tunable devices that can correct for many more modes without added bulk. This technique seeks to manipulate the refractive index of a nematic LC by selectively polymerizing the LC through the addition of monomers that fix the orientation of the director field (the director being the average pointing direction of the long axes of the molecules), regardless of an externally applied electric field. Previous explorations have generally relied on relatively low-resolution manufacturing methods, such as the use of photomasks^10−12^ or laser beams with a predetermined spatial profile of the intensity.^13^ While these techniques are sufficient for manufacturing a relatively simple lens shape such as a conventional planoconvex lens, they are typically insufficient for more complex devices.

**Figure 1:**
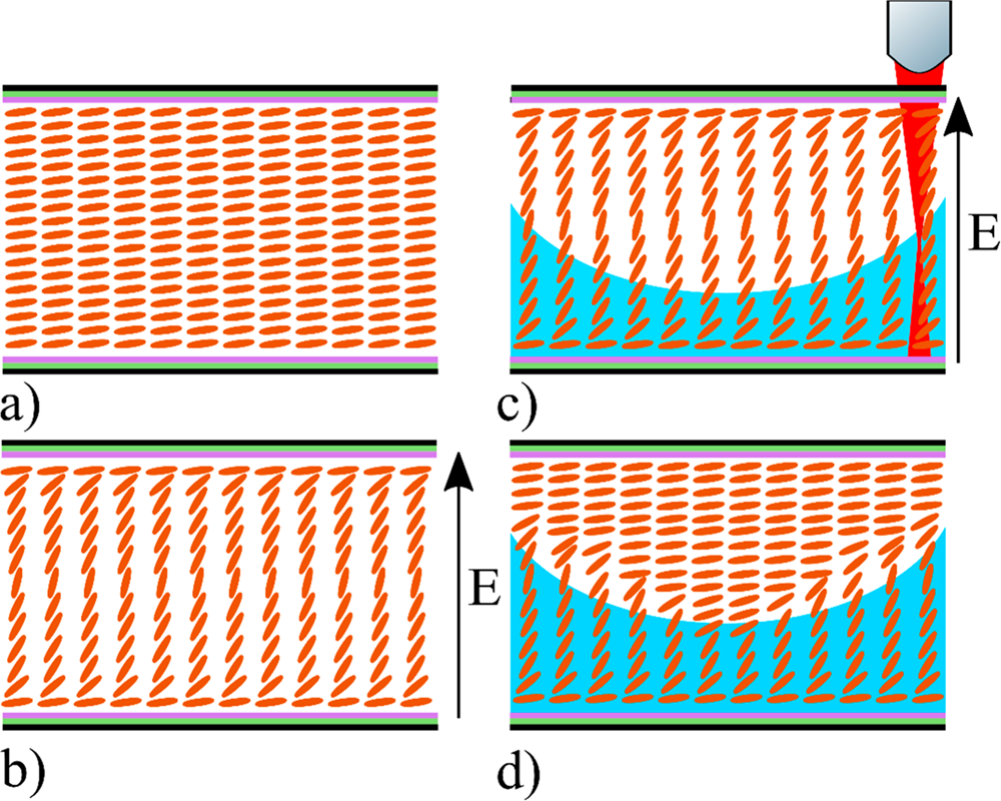
Illustration of the laser writing process of a nematic LC designed to correct for an aberration mode. (a) Nematic LC glass cell in the absence of an applied electric field, with the LC director orientation shown in orange, the polyimide alignment layer shown in purple, and the indium tin oxide (ITO) coating shown in green. (b) The same LC with a large amplitude electric field applied; the director is now oriented perpendicular to the substrate in the bulk of the device. (c) Laser writing process at a large amplitude electric field, with the laser shown in red and the polymer network shown in blue. (d) Nematic LC after the electric field has been removed and the laser writing process has finished. Here the polymerized regions (shown in blue) preserve the director field profile of the switched state.

In recent years, direct laser writing (DLW) has enabled the manufacturing of high-resolution polymer structures directly within a liquid crystalline mixture.^14−20^ Though direct laser writing in photoresist laid the groundwork for these processes,^18^ writing in LC instead has significantly improved the efficiency of the system. Here, by mixing the LC with a monomer and photoinitiator, the LC mixture is selectively polymerized using a femtosecond laser that triggers polymerization through a two-photon absorption process. By scanning the focus relative to the LC, this allows for high spatial resolution selective polymerization in three dimensions within the LC layer. The polymerization process permanently fixes the LC director field of the illuminated area at a particular orientation at the point of exposure. This fixed angle of the director can be further tuned by adjusting the applied voltage during the writing process. A diagram of this design principle is shown in Figure 1. In this paper, we present a series of devices that have been fabricated with DLW to create a polymerized structure to correct for a single Zernike mode that can be subsequently controlled by the application of an electric field. The devices can correct such a mode with continuous gray scale tuning, to a total phase difference of more than 2π radians peak to peak.

## Results and Discussion

### Design of the Modal Correctors

**Figure 2:**
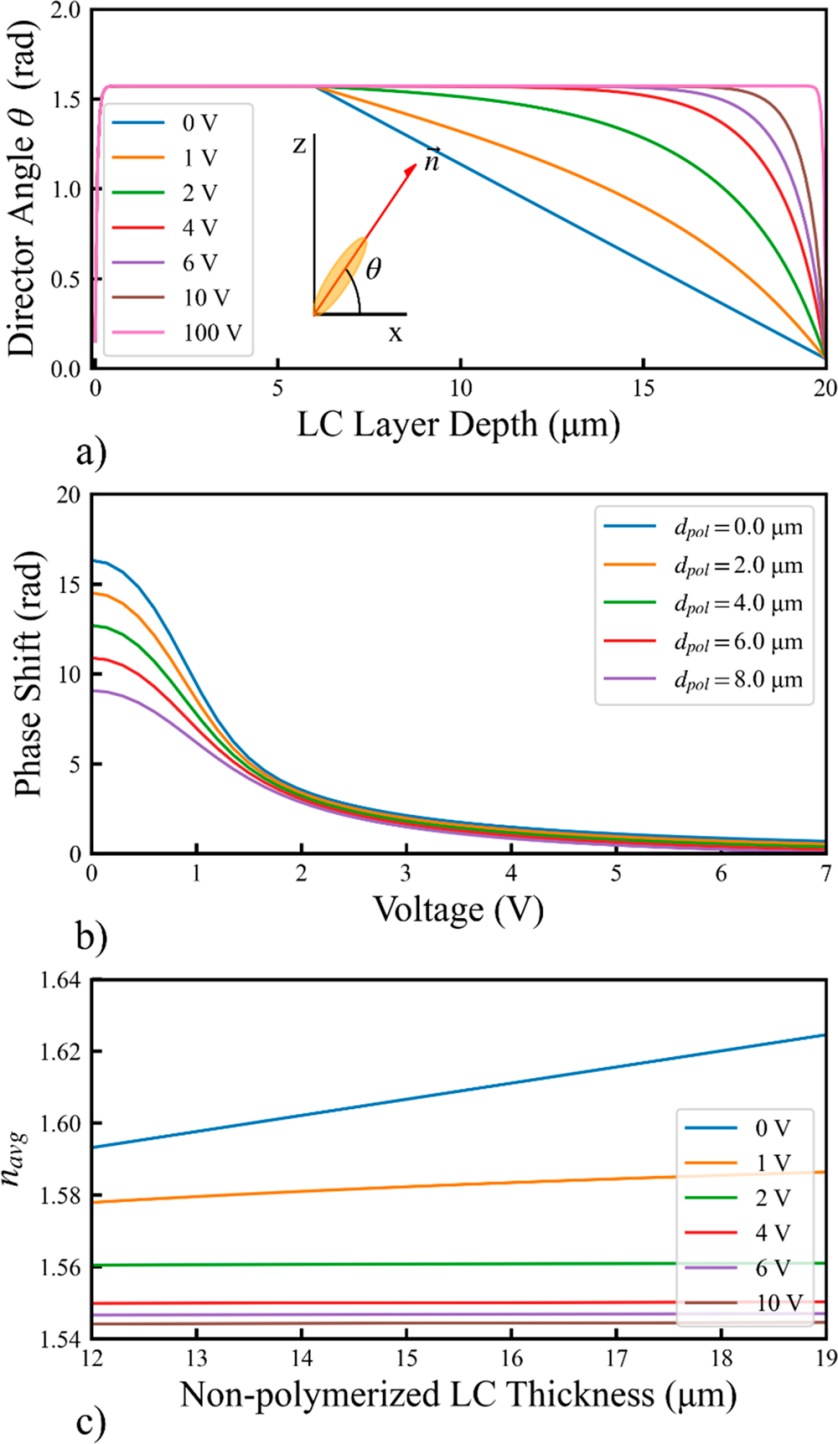
(a) Simulated LC director profile when a voltage of 100 V was applied to the nematic LC with a layer thickness of 20 μm. Also shown are the director profiles for a variety of voltages for a polymerization depth across the LC layer of 6 μm. The inset illustrates the definition of the director angle, θ, where the substrate is parallel to the *x*-axis with light propagating along the *z*-axis (the director *n⃗* is shown in red for a longitudinal LC molecule (orange)). (b) Relative phase shift through the nematic LC device for various polymerization thicknesses *d*_pol_. (c) Simulated *n*_avg_ (as defined in eq 2) of a 20 μm cell, relative to various thicknesses of nonpolymerized LC throughout the depth of the device and at various voltages.

The modal correction devices were manufactured in LC glass cells with planar alignment layers and transparent electrodes and were capillary filled with a mixture consisting of 79 wt % nematic LC (E7), 20 wt % reactive mesogen (RM257), and 1 wt % photoinitiator (IR819; see Methods). Laser writing was conducted at a voltage of 100 V to ensure a homeotropic LC alignment of the director during exposure to the laser writing process. Such a voltage also ensured that the influence of the optical field of the laser, which could impose a torque on the director, was minimal.^21^ Before fabrication, devices were carefully designed with the aid of simulations of the director profile of the nonpolymerized LC at a voltage of 100 V. This director profile, along with that for a mode corrector with a polymerization height of 6 μm, is shown in Figure 2a. In order to ensure a uniform director field boundary condition within the cell of θ_substrate_ = 0° and θ_polymer_surface_ = 90°, a minimum polymerization thickness of 1 μm was selected for the device. When the LC is confined between two glass substrates, with a depth dependent director angle θ(*z*) formed relative to the substrate (as illustrated in the inset in Figure 2a), the refractive index for light polarized along the director axis is defined as
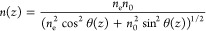
1where *n*_e_ is the extraordinary refractive index, which is parallel to the optic axis of the nematic LC and *n*_0_ is the ordinary refractive index, which is perpendicular to the optic axis. A description of the simulation methodology employed in this work can be found in Methods.

The relative phase shifts induced by the device at various polymerization thicknesses and voltages were simulated, as shown in Figure 2b. These simulation thicknesses were selected as the total polymerization thickness was expected to vary from 1 μm to a maximum height of 8 μm (which is approximately the maximum polymerization thickness achievable by the DLW system in a single pass and is governed by the laser focus size). As expected, this device induced a maximum phase shift when the applied voltage to the device was set to 0 V. From here, we determined the average refractive index of the device at this design voltage for various polymerization thicknesses, as shown in Figure 2c, along with the average refractive index of the device at higher voltages to better predict the performance of the device at intermediate voltages. The average refractive index is defined as
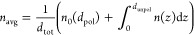
2where *d*_tot_ is the total thickness of the LC layer, *d*_pol_ is the thickness of the laser-induced polymerization region, and *d*_unpol_ is the remaining nonpolymerized LC region, which is *d*_unpol_ = *d*_tot_ – *d*_pol_. The polymerization thickness of the device was selected by relating the phase profile of the desired Zernike mode *Z*(*x*,*y*) with the final polymerization thickness through the relation,
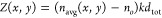
3where *k* is the wavenumber (the corresponding wavelength was selected to be 660 nm for the set of devices considered in this work), and the final polymerization thickness was related to the desired *n*_avg_ through eq 2.

### Fabrication and Demonstration of the Modal Correctors

**Figure 3:**
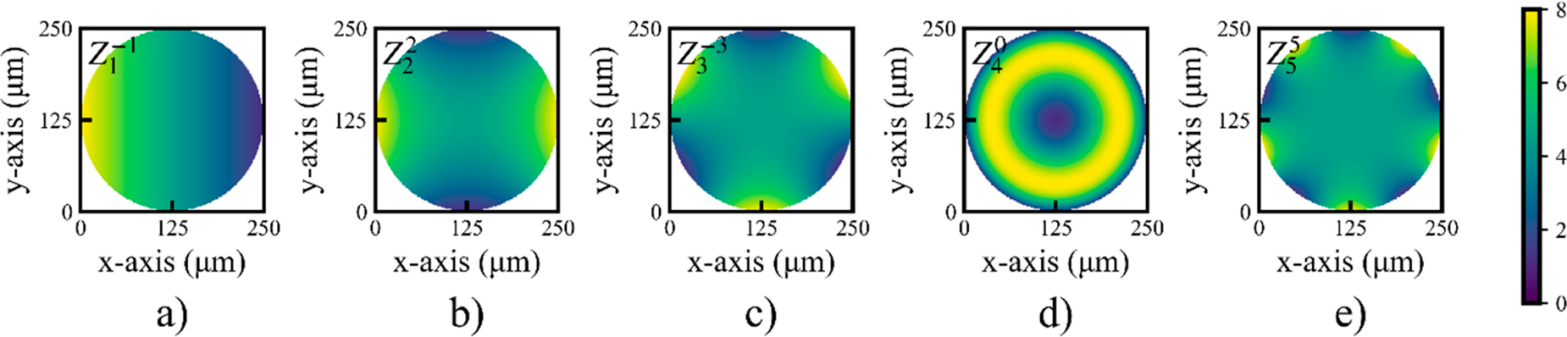
Ideal polymerization design thickness (μm) for the five different Zernike modal correctors, with color corresponding to thickness ((μm) as described by the color bar on the right): (a) tilt (*Z*_1_^–1^), (b) astigmatism (*Z*_2_^2^), (c) trefoil (*Z*_3_^–3^), (d) spherical (*Z*_4_^0^), and (e) pentafoil (*Z*_5_^5^).

Five devices were manufactured. Using the simulated curve shown in Figure 2c and eq 3, we calculated the ideal polymerization thickness required for five Zernike modes, corresponding to tilt, astigmatism, trefoil, spherical, and pentafoil (*Z*_1_^–1^, *Z*_2_^2^, *Z*_3_^–3^, *Z*_4_^0^, and *Z*_5_^5^, respectively). These devices were designed such that the maximum phase difference across the device was a total peak–peak amplitude of 2π radians. The ideal design polymerization thicknesses of these devices are shown in Figure 3 for modal correctors measuring 250 μm in diameter. These devices were designed so that the maximum polymerization thickness of the devices, illustrated via the color bar on the right, did not exceed 8 μm, the maximum single pass polymerization height of the writing apparatus. Since these devices were designed to be manufactured when a very high voltage was applied to the LC bulk, a higher polymerization height generally corresponds to a lower total phase shift, as the director field of the polymerized bulk should be nearly entirely oriented at θ = 90° and, thus, display an average refractive index *n*_pol_ = *n*_0_.

**Figure 4:**
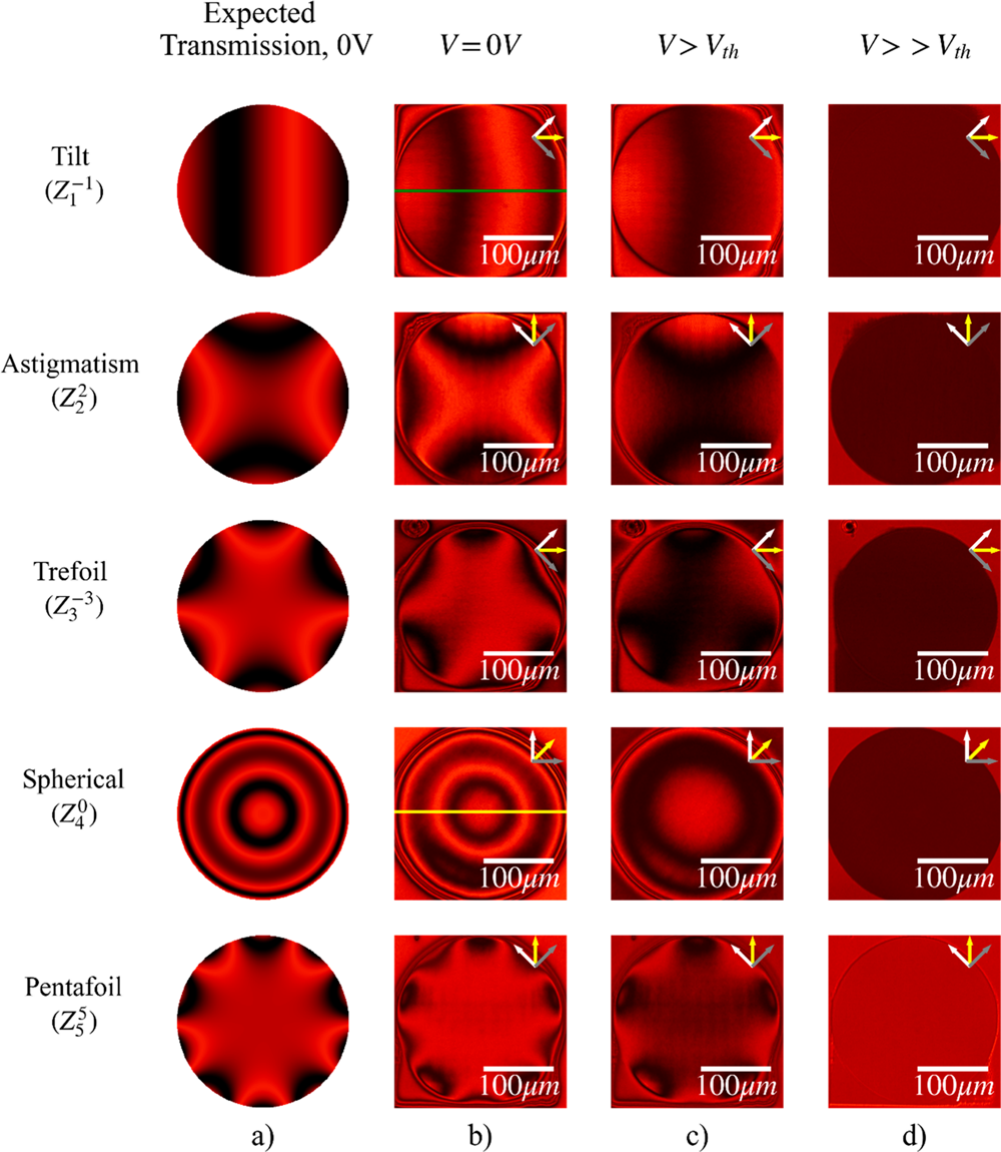
Simulations and experimental results for five Zernike mode LC correctors (*Z*_1_^–1^, *Z*_2_^2^, *Z*_3_^–3^, *Z*_4_^0^, *Z*_5_^5^) for continuous gray scale mode correction, manufactured in a 20 μm thick cell. Images were taken on a polarizing optical microscope at various voltages, measured at a temperature of approximately 20 °C. While the modes were manufactured at different orientations relative to the rubbing direction, this does not affect the performance of the device. (a) Expected (simulated) transmission of each of the five Zernike modes when viewed on a polarizing optical microscope, (b) experimental results of the different devices operating at 0 V, (c) operating above the Fréedericksz threshold voltage *V*_th_, at a value between 1.2 to 2.0 V, and (d) at a higher voltage of 10 V. The gray and white single-headed arrows indicate the polarizer and analyzer directions, respectively, whose transmission axes were crossed, while the yellow single-headed arrows indicate the rubbing direction of the alignment layers.

The devices were manufactured using the DLW system, as described in the Methods, and imaged using polarizing optical microscopy (POM). Figure 4 shows the expected transmission of the desired Zernike mode through crossed polarizers for a wavelength of 660 nm (Figure 4a), along with the corresponding experimental results for the manufactured modal correctors at three different representative voltages: no voltage applied (Figure 4b), a voltage above the Fréedericksz threshold voltage (Figure 4c) of E7, and a voltage that is significantly greater than the Fréedericksz threshold voltage (Figure 4d) of E7. When no voltage was applied (Figure 4b), the director field for the nonpolymerized LC was parallel to the substrate and the average refractive index for this region was *n*_unpol_ ≈ *n*_e_. However, when the applied voltage was just above the Freederickz threshold voltage, the director field was instead oriented at angles between 0° and 90°, and thus the nonpolymerized LC had an average refractive index between *n*_0_ and *n*_e_, as described by eq 1. When a voltage significantly higher than the Freederickz threshold voltage was applied, the director field of the LC was aligned to the normal of the glass substrates, with the average refractive index given by *n*_avg_ ≈ *n*_0_, thus becoming uniform with the refractive index of the polymerized bulk of the LC *n*_pol_ ≈ *n*_0_. The transition voltage was around 1.2 to 2.0 V, depending on the imaged device, while 10 V was the highest voltage applied to the devices after fabrication. The simulated transmission for each mode shows very good agreement with the POM images obtained in the absence of an applied voltage. These devices can be readily adapted to provide wavefront tuning for any monochromatic light source across a broad range of wavelengths, with only voltage amplitude adjustments required to ensure the correct phase profile for the desired operational wavelength. However, like many phase correction sources, the performance would still be limited for broadband wavefront sources, which is something that we hope to consider in future studies along with a refinement of the mixture formulation to ensure that the devices can be operated at shorter wavelengths of illumination (e.g. <450 nm) to avoid unwanted cross-linking of any remaining reactive mesogens.

**Figure 5:**
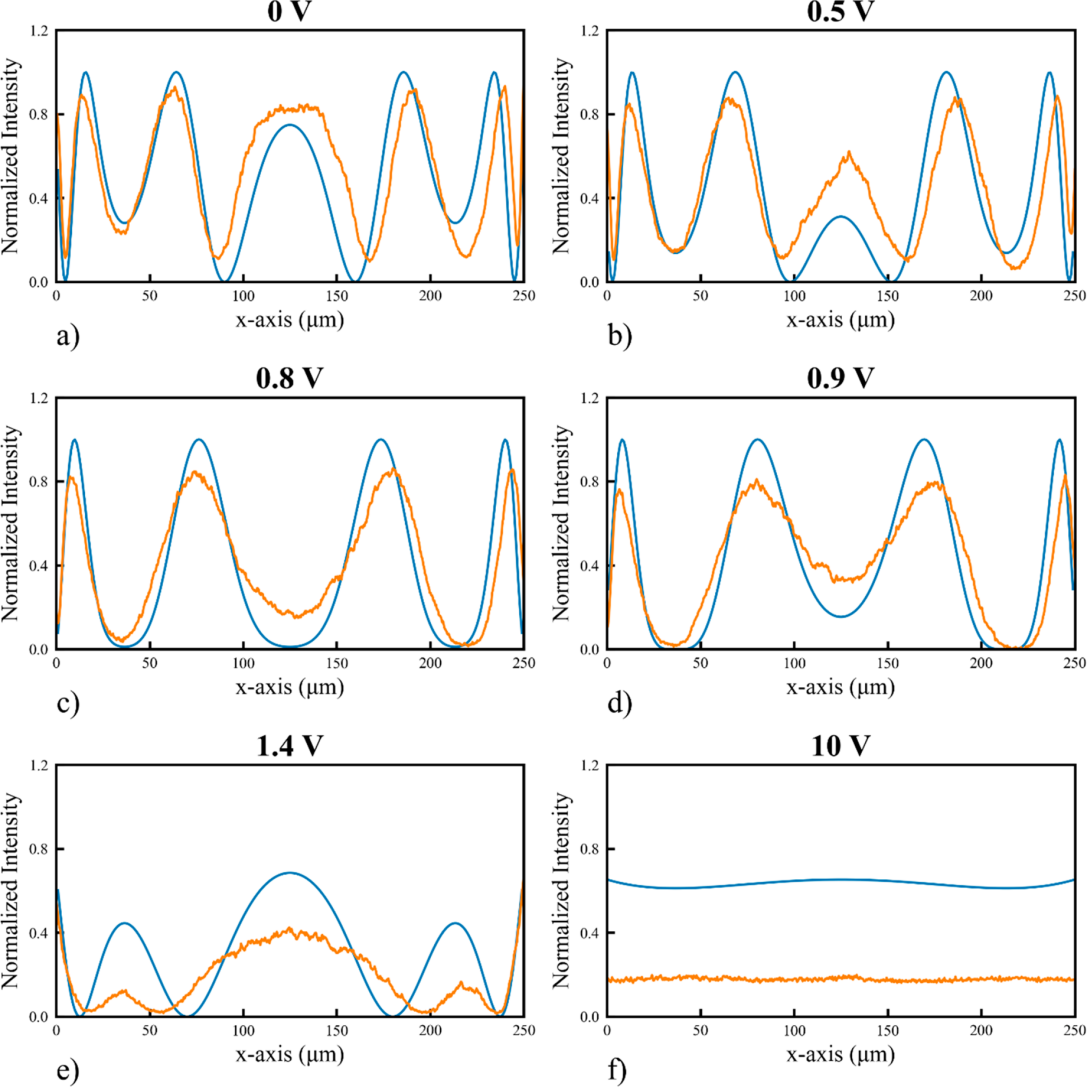
Measurements (orange line) of the normalized intensity of the transmitted light for the LC Spherical Mode Corrector (*Z*_4_^0^) at six separate voltages of (a) 0, (b) 0.5, (c) 0.8, (d) 0.9, (e) 1.4, and (f) 10 V. Intensity is proportional to the retardance through the relation *I* = sin^2^(ΔΦ/2), where ΔΦ is the retardance. Results from simulations are shown as the blue solid line. In both cases, the results are obtained from the transmission through the LC mode corrector along the yellow line in Figure 4b parallel to the *x*-axis, as defined in Figure 3, when it is between crossed polarizers for incident light of wavelength 660 nm.

To compare our experimental results with simulations in more detail, the spherical Zernike mode (*Z*_4_^0^) was analyzed further for various applied voltages, as shown in Figure 5. In this figure, we compare cross sections of the normalized intensity measurements from the POM images for the spherical mode device with the predicted transmission of the device at each of the demonstrative voltages. Here, the design polymer thickness was equivalent to that demonstrated in Figure 3d. The details of the simulation method used to predict the expected transmission can be found in Methods. The experimental results were obtained by sampling along the yellow line shown in Figure 4b as the amplitude of the applied voltage was increased. These samples of the normalized transmission were taken at 0, 0.5, 0.8, 0.9, 1.4, and 10.0 V for Figure 5a–f, respectively, demonstrating the change in transmission of the modal corrector as the voltage was increased. These plots show good agreement between the normalized transmitted intensity and the transmitted intensity predicted from simulations of the corrector. These results indicate that the manufactured polymer thickness cross-section indeed agrees well with the intended polymer thickness required to create the spherical modal corrector (*Z*_4_^0^). The slight differences between the observed and predicted transmission in Figure 5e,f (higher voltages) are likely the result of a combination of inaccuracies in the one elastic constant approximation used for the simulation (described in full in Methods) coupled with small errors in the estimation of the “zero height” of the writing process during manufacturing, which introduced a constant offset to the polymerization height and therefore the total retardance.

**Figure 6:**
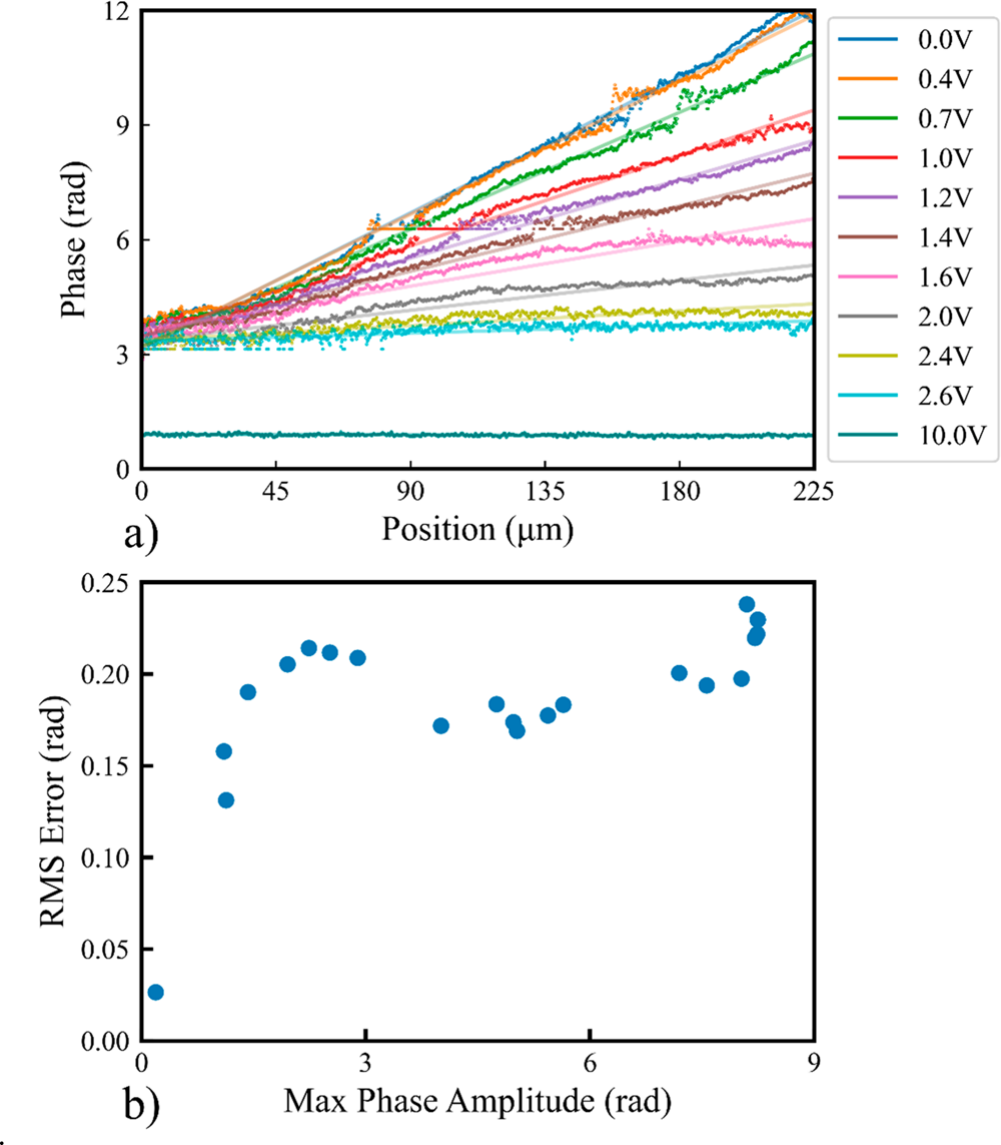
(a) Estimated phase of the tilt modal corrector at various voltages, compared to the ideal tilt profile shown by the solid line of the same color, imaged along the green line shown in Figure 4b. As observed, the total phase amplitude of the mode corrector decreases as the total voltage is increased. (b) RMS error of the estimated phase along the line compared to the ideal tilt profile, relative to the total amplitude of the tilt phase profile.

By comparing the expected transmission of an ideal tilt mode to the measured transmission, we were also able to further determine the phase through the tilt correcting lens shown in Figure 4, sampled along the green line in the same figure. Since the fast axis of the LC device is held constant, the retardance of the device is a good measure of the relative phase shift traveling through different portions of the device. The result is shown in Figure 6a, along with the ideal tilt profile at the same voltage. While the entire device measured 250 μm across, we considered only the central 225 μm to be the active aperture of the device during these phase estimates and so only measure the intensity across this portion of the device. A full description of the phase estimation method is available in the Methods. As can be observed in the figure, the phase profile across the lens remains roughly linear across it, with the peak-to-peak amplitude of the lens starting at over 2π radians peak to peak at 0 V, and slowly decreasing as the voltage is increased. At 10 V, the phase across the lens is almost entirely constant, although the voltage still allows for a small amount of retardance between the fast and slow axis.

To better verify the accuracy of our lenses, Figure 6b presents the RMS error value of the phase estimates presented in Figure 6a with a tilt mode (*Z*_1_^–1^) profile of identical magnitude. The RMS error was calculated as the RMS difference between the estimated phase values and the ideal phase at that voltage, shown via the solid line of the same color in Figure 6a. An exact definition of the RMS error is presented in Methods. The max RMS error between the measured phase and the expected tilt (*Z*_1_^–1^) along the same axis was approximately 0.25 rad, while the minimum was 0.03 rad. Aside from the transparent state, the RMS phase error in the lens hovered roughly around 0.20 rad. For very high voltages, where the maximum phase difference across the lens is 0, the error became negligible. For high modal magnitudes, these errors are largely introduced by edge effects at the extreme ends of the lens. For intermediate modal magnitudes, the director orientation of the nonpolymerized LC region was no longer relatively uniform, resulting in a nonlinear phase behavior. This can be observed in Figure 6a, in the line corresponding to *V* = 1.6 V.

**Figure 7:**
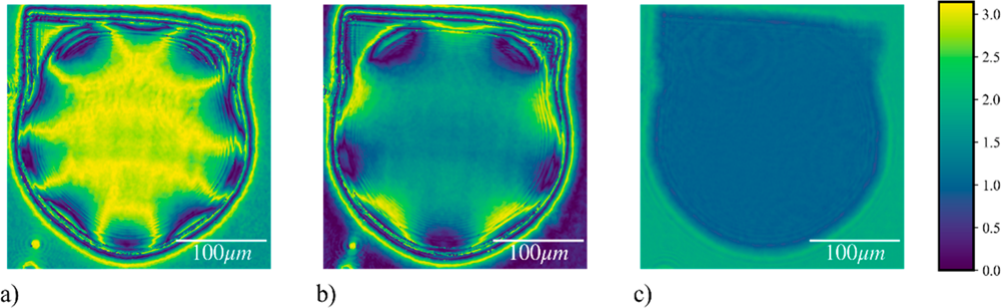
Retardance images of a laser-written LC pentafoil Mode Corrector obtained at (a) 0, (b) 1.0, and (c) 10.0 V, with retardance indicated via the color bar on right in radians.

To further confirm our results, retardance images of the pentafoil device (*Z*_5_^5^) are shown in Figure 7, which were decomposed^22^ from the Mueller matrix images recorded by an imaging polarimeter.^23^Figure 7a–c shows the device imaged at 0, 1.0, and 10 V, respectively. The retardance images provide a quantitative measure of the distribution of the birefringence value of the devices. Additional vectorial metrics^23^ can also be extracted from Mueller matrices. Note that the decomposition method^22^ confines the retardance value to the region of [0,π]. However, from the observation of the retardance value along the edge of the devices in Figures 7a,b along with prior knowledge regarding the configuration of the devices, it is straightforward to infer the regions for which retardance value is from [π,2π] because they are expected to vary linearly with the voltage.

## Conclusions

We have presented a novel liquid-crystal-based aberration correction device that offers a versatile manufacturing method that can be adapted to multiple modes. To illustrate the potential of this approach in generating a range of complex modes, we have presented demonstrator devices corresponding to the five Zernike modes of tilt (*Z*_1_^–1^), astigmatism (*Z*_2_^2^), trefoil (*Z*_3_^–3^), spherical (*Z*_4_^0^), and pentafoil (*Z*_5_^5^). Each device is optically transparent for easy integration into the optical path and can correct for a single Zernike mode with continuous grayscale tuning to a maximum phase difference magnitude of over 2π radians peak to peak. The devices are electronically very simple, being operated by a single electrode pair tunable between 0 and 10 V. Experimental results indicate that the devices have been manufactured to a high accuracy, with the tilt device displaying a maximum RMS phase error of 0.25 rad. This accuracy has been verified as well through direct phase imaging of the pentafoil mode (*Z*_5_^5^), as well as through a comparison between the expected (simulated) transmission of the spherical mode corrector (*Z*_4_^0^) between crossed polarizers and the actual measured transmission. With our current laser writing facility, the minimum feature size accessible is 0.7 μm, although the impact of diffusion of the polymer network and the continuous nature of the director field are also likely to impact the final resolution of the writing process. The demonstration devices here measured only 250 μm in diameter, as they were manufactured as proofs of principle, but in the future it would be possible to manufacture correctors across a larger area and so accommodate much more complex phase patterns. We therefore believe that the fabrication methodology presented in this work could be applied to the generation of any other arbitrary mode, limited only by the precision of the laser fabrication and the physical properties of the LC mixture.

## Methods

### Simulations

The laser-written modal correctors were designed using a finite difference director field simulation that employed a one constant approximation of the elastic constant and a variable electric field amplitude.^24^ Each iteration of the finite difference solver was updated using the difference equation,
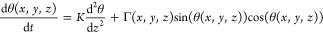
4where *K* is the one constant approximation, selected as 1 × 10^–11^ N for the nematic LC (E7), and Γ is the spatially varied dielectric coupling term calculated as Γ(*x*,*y*,*z*) = Δϵϵ_0_*E*(*x*,*y*,*z*)^2^, where *E* is the local electric field. The spatially dependent electric field was, in turn, calculated from the director field. First, the dielectric permittivity along the *z*-direction of the nonpolymerized LC can be expressed as
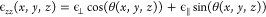
5while the dielectric permittivity of the polymerized LC was approximated to be constant at
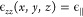
6Thus, keeping in mind that the dielectric displacement, *D* = ϵ*E*, is constant given a charge-free material and that the electric field and the total electric field must be related to the applied voltage through *V* = ∫_0_^*d*_tot_^*E*(*z*)d*z*, we can calculate the electric field at any location as
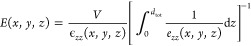
7Solving the director and electric field iteratively allows the LC director profile to be determined. Once the director has been calculated, the total phase shift can then be calculated as
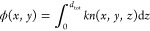
8where the local refractive index is calculated through the relation
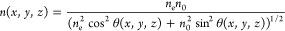
9If the total transmission between cross polarizers is required, we note that the transmission of the liquid crystal device can be expressed as
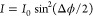
10where Δϕ is the difference in phase between the slow and fast axis of the LC cell. Noting that the slow axis has a uniform refractive index of *n*_0_, we can thus rewrite this equation as
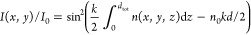
11where *n*(*x*,*y*,*z*) is related to the simulated director field through eq 9.

### Materials

The devices were manufactured in Instec LC2-20 glass cells with 20 μm spacers. These cells consist of two parallel glass plates, with the inner surface coated with transparent indium tin oxide electrodes and an antiparallel rubbed polyimide alignment layer. These cells were filled with an LC mixture consisting of 79 wt % E7 (Synthon Chemicals Ltd.), with 1.0 wt % Irgacure 819 (Ciba-Geigy) photoinitiator and 20 wt % RM257 (1,4-bis-[4-(3-acryloyloxypropyloxy) benzoyloxy]-2-methylbenzene (Synthon Chemicals Ltd.) reactive mesogen. This mixture formulation was used in this study, as it forms a nematic LC phase at room temperature and has previously been shown to be compatible with the laser writing process. Two wires were soldered to the two surface electrodes of the LC cell using an indium solder.

### Laser Writing Process

The cell was then mounted on a glass microscope slide using UV-cured glue before being filled with the LC mixture through capillary action. This slide was then exposed to a Spectra Physics Mai-Tai Titanium-Sapphire laser (λ = 780 nm) providing 100 fs pulses at an 80 MHz repetition rate. The laser was focused with a 0.45NA objective lens with 20× magnification. The sample was placed on a 3D translation stage built by combining an Aerotech ANT95XY 2D translation stage with an ANT95v vertical translation stage. This exposure was done while a square wave with a peak to peak voltage of 100 V was applied to the cell at 1 kHz, using a Tektronix AFG3021 signal generator and an FLC F10AD amplifier. The 3D translation stage allowed us to move the LC cell in a raster pattern, adjusting the height continuously during the writing process with a height determined via eq 3. This pattern was written with a pixel size of approximately 0.5 μm and a nominal writing speed of 1.25 mm/sec.

### Polarizing Optical Microscope Characterization

Each modal corrector was imaged through crossed polarizers using an Olympus BX51 polarizing optical microscope and a QImaging R6 Retiga Camera with an Olympus LMPLFLN20x objective lens for 20× magnification. To prevent further polymerization and ensure narrow band retardance imaging, a narrowband Thorlabs FB660-10 filter was mounted in the illumination path. The devices were connected to a Multicomp MP750510 AC square wave signal generator, with a frequency of 1 kHz and imaged at various peak to peak voltages between 0 and 10 V.

### Estimating the Phase Profile

For any birefringent material, the intensity transmitted through the device located between crossed polarizers with a fast axis oriented at 45°, *I*, is proportional to the initial intensity of the light source *I*_0_ through the relation,
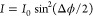
12where Δϕ is the difference in phase between the fast and slow axis, which for the LC is thus
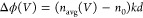
13where the calculation of *n*_avg_(*V*) is discussed above. Because of the sin^2^ relation, every detected intensity had the potential to be generated by several different phases. Thus, seven potential phase arrays were generated for each intensity. First, by normalizing the detected intensity and then defining θ(*x*) = 2 arcsin√*I*(*x*), we generated a potential phase matrix *M* such that
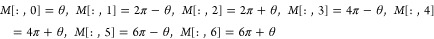
14where *I*(*x*) is the pixel intensity measured along the center axis of the device. As we had already determined through simulation that it was very likely that the polymer thickness was correctly manufactured, we could assume that the phase measured across the lens was roughly monotonically increasing. As such, we assumed that at *x* = 0 in the lens, the retardance observed Δϕ(*x* = 0) = *M*[0,1]. From here, we continued matching the intensity of each pixel *I*(*x*) with its corresponding expected phase value *M*[*x*,*j*] for increasing values of *x*, where *j* is the phase estimation variable that begins with *j* = 1. Finally, we define a threshold δ such that if *M*[*x*,*j*] – *M*[*x*,*j* + 1] < δ, then the integer *j* will be incremented by 1 for all remaining values of *x*. This process is repeated until the data set is exhausted. The total RMS error was defined as
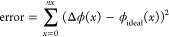
15where Δϕ(*x*) is the estimated phase of across lens calculated with this method, and ϕ_ideal_(*x*) is the closest linear fit to this phase profile calculated with a least-squares fitting method.

### Mueller Matrix Polarimeter

To confirm our results, the pentafoil mode corrector (*Z*_5_^5^) was imaged using a Mueller matrix polarimeter.^18^ These measurements provide confirmation of the device functionality when the device was tuned to an intermediate voltage *V* > *V*_th_, as the device still exhibited the appropriate retardance profile to that of the desired Zernike mode and also allowed us to infer the functionality of the device at 0 V, as the retardance profile of the device was expected to vary linearly with voltage.
